# A novel rat head gaze determination system based on optomotor responses

**DOI:** 10.1371/journal.pone.0176633

**Published:** 2017-04-26

**Authors:** Mengbo You, Toshiyuki Yamane, Hiroshi Tomita, Eriko Sugano, Takuya Akashi

**Affiliations:** 1 Department of Design and Media Technology, Graduate School of Engineering, Iwate University, Morioka, Iwate, Japan; 2 Department of Chemistry and Biological Sciences, Faculty of Science and Engineering, Iwate University, Morioka, Iwate, Japan; Dalhousie University, CANADA

## Abstract

The optomotor response of animals is commonly used to measure their visual performance, e.g., rats of different genetically altered strains or various drug tests. With the presentation of stimuli using computer screens or projectors, the common idea focuses on measuring the eye movement or head and/or body movement to characterize changes of the head gaze. However, traditional methods rely on either the invasive fixation of animals, or the judgment of a human observer who reports the stimulus-tracking movements. In this paper, we propose a novel head gaze determination system to automatically track the head movement of rats without artificial markers. The experiments were done to demonstrate the process of optimizing parameters in image processing. As a result, the head angle curve of the proposed method is consistent with that of ground-truth data annotated manually according to predefined rules. Hence, the proposed method provides a simple, convenient, and objective solution to automatically generate the head gaze orientations from massive amounts of recorded data for further visual performance analysis.

## Introduction

Mouse genetics has a longstanding tradition in behavioral analysis [1]. Even though mice and rats are nocturnal and navigate largely with their noses and whiskers, they are frequently used in biological and biomedical laboratories to study the function and diseases of the visual system. The reason lies in the availability of genetically diverse inbred strains and the ease of generating single-gene mutations to study the mouse’s behavior using both forward and reverse genetics [2]. Moreover, it is easier to investigate how the brain processes visual stimuli at the cellular level on mice than on primates [3]. A large variety of behavioral tests [4] has been developed to assess various properties of the rodent vision system, which is responsible for accomplishing many complex tasks, from tracking moving targets to discerning spatial details or specific color patterns in the object being viewed.

Visual properties, e.g., contrast thresholds, spectral sensitivities and spatial or temporal acuity, are frequently measured utilizing the optokinetic response (OKR) [5] or the optomotor response (OMR) [6] [7] [8]. The OKR is an eye movement driven by certain visual stimuli, which can be observed by watching the pupil. The OMR is a distinct involuntary movement of the body or head, which has proved to be robust and reproducible in various mouse strains under either scotopic or photopic conditions [9]. Both responses are triggered to stabilize the image on the retina when an animal visually perceives the movement of large parts of the visual environment. The measurement of these reflexes is particularly used in mice [4] [9] [10] [11] to characterize the visual performance of different mouse strains or drug effects. In this study, we developed a novel head gaze determination system for visual ability analysis based on the OMR.

OptoMotry [12], a commercially available virtual optomotor system for quantifying the visual threshold, has been widely used to measure the spatial vision of freely moving mice [13] [14] [15]. This system utilizes four computer monitors facing each other to form a cube, in which a rotating cylinder with a vertical sine wave grating is calculated and drawn on the inner surface of the cube. While this device serves as a useful tool to measure parameters of the visual system, the head movement in response to large scale visual stimuli, was measured with only a rough estimate to determine the existence of induced optomotor response and never systematically analyzed. A previous literature [16] revealed that the reason might be the lack of a suitable video tracking algorithm to determine the head and body angle precisely enough during an experiment. They also proposed a new tracking algorithm based on the contour and areas of binary objects which is segmented by applying a range threshold. However, their device used LED strips attached in all four edges and mirrors on both bottom and top side of their setup to provide gratings inside a virtual sphere surrounding the animal. The capture range contains the animal, a small platform supporting the animal, a large square area of the bottom mirror reflecting gratings on four computer screens. The boundary between the animal body region and the changeable grating region is not clear enough to extract contour correctly. The literature demonstrates that only in the case of an albino animal at scotopic light condition with infrared filter, their method succeeded to detect the contour. However, their method failed to detect the mouse contours correctly in the other three cases:

- The mouse with agouti coat color at photopic light condition;
- The black coated animal at photopic light condition with infrared filter;
- The black coated animal at scotopic light condition with infrared filter.

In this study, we apply a similar variant of the OptoMotry apparatus that uses a projector to provide a wider adjustable illuminance range and develop a set of new body-sensing and behavior-analysis methodologies. The apparatus is designed to provide the grating stimulus with a yaw range of 90°. The bottom and surrounded panel can be replaced by materials with less refection to reduce noise while detecting contour. In this paper, we used metal panels of dark colors to make a clear color difference between the animal region and other regions. For non-albino animals, the panels can be replaced by other materials of different color and absorption properties, corresponding to the coat color. By this way, our solution is supposed to detect animal contour of different coat colors successfully. The length of bristle also has little influence on the proposed method.

## Methods

### Stimulation and recording

**Fig 1:**
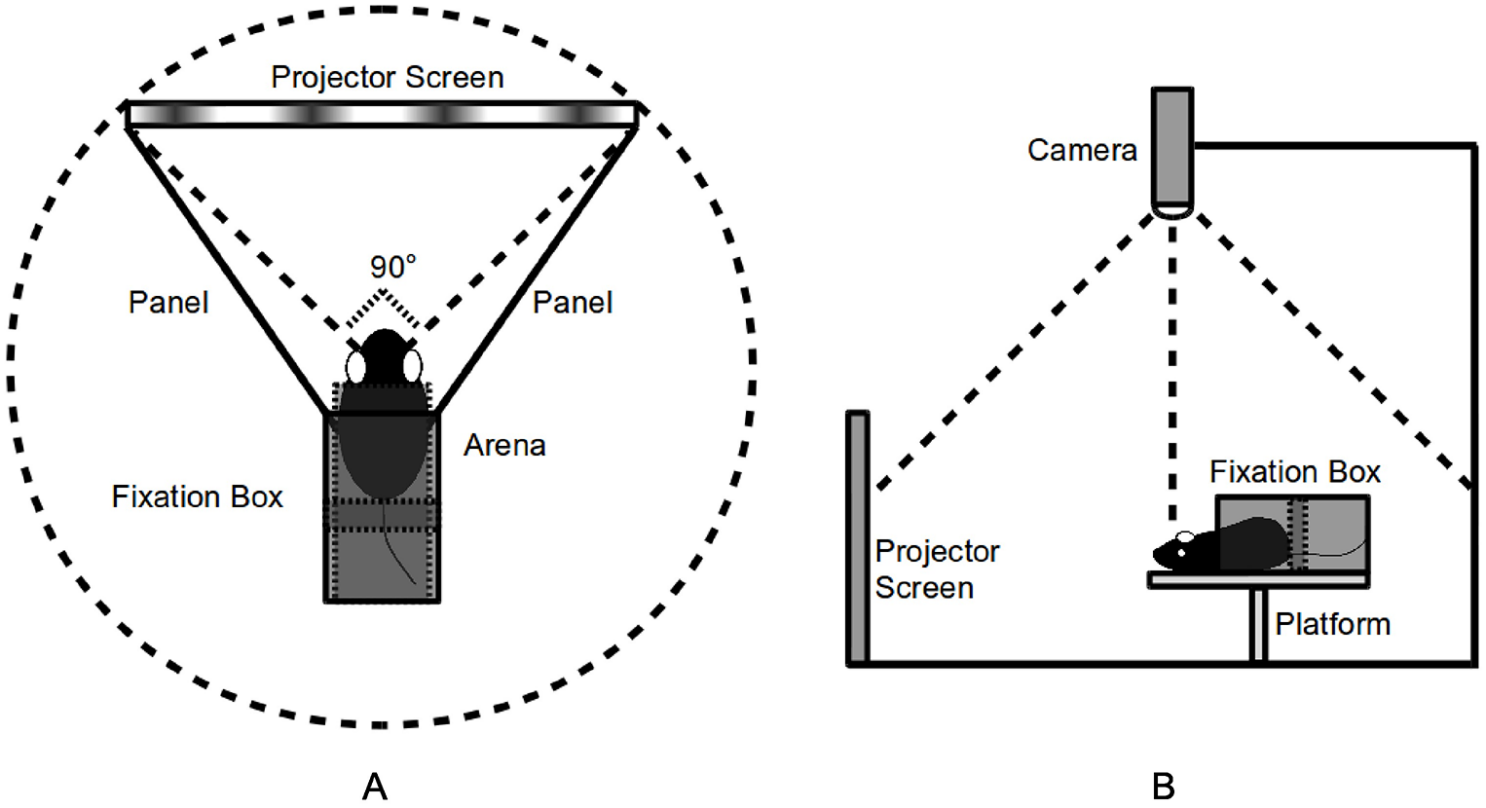
Overview of the apparatus to display the stimulus and record the response behavior. A projector connected to a laptop PC is utilized to present the stimulus on the screen. However, the projector and laptop are omitted in this figure to focus on the environment of testing subject. A: Top view. B: Side view.

Unlike the OptoMotry apparatus, we utilize a projector (BenQ MX764) and projection screen to present the stimulus. We choose a projector rather than a monitor because the computer monitor is normally designed to be dim to protect human eyes. Its limited adjustable screen luminance range is unsuitable for accurately measuring rats with poor visual ability. The testing arena is surrounded by a screen, a pair of symmetric metal panels, and a fixation box (Fig 1A). The fixation box does not immobilize the body, but uses a rectangular backup bar to prevent the rat from moving backwards. The platform is longer than the fixation box to guarantee sufficient action space. The screen blocks about a 90° arc in the virtual cylinder. The rotating circular stimulus is projected onto the straight screen. The response behavior is recorded by a camera (Optitrack Slim3U) set on top of the platform with an adjustable height (Fig 1B).

The rats are put on the platform and surrounded by the fixation box for 15∼30 minutes in total for every experiment day. Between two experiment days, the period without any experiments lasts about 2 weeks∼1 month. In each experiment day, the maximum number of times for stimulus test is 12. Each test lasts 3 minutes in maximum. Between two tests, there is a break of 2∼5 minutes.

### Stimulation software

**Fig 2:**
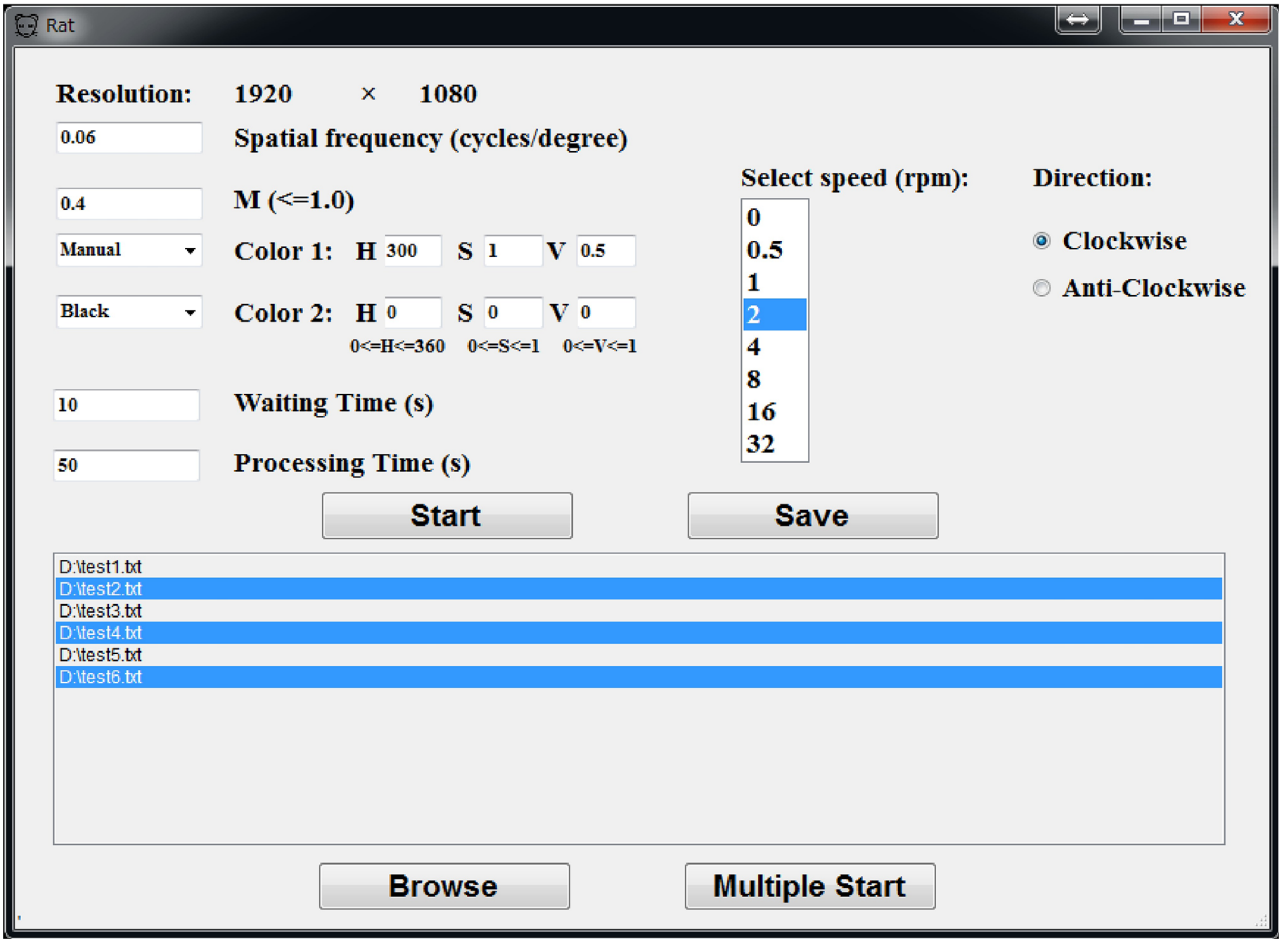
Stimulation software interface. This software provides free definition of two adjacent color for the gratings, precise control of waiting time and processing time. Moreover, it can save parameter setting into text files and start multiple stimulations by simultaneous multiple reloads, which is efficient and avoids distraction of the animal while changing parameters.

The stimulation software draws the stimulus on the virtual cylinder, which rotates according to the pre-defined speed. The observation screen accounts for about a quarter of the cylinder. The stimulus parameters that can be specified before the OMR experiment (Fig 2) include the spatial frequency, neighboring color pair, varying intensity of the stimulus pattern, waiting time (time before the rotating stimulus is presented), processing time (time that the rotating stimulus is presented), rotating speed, and rotating direction. Furthermore, several sets of parameters demonstrating different types of experiments can be saved as text files and loaded to execute continuously without a break, to avoid any possible gradual distraction of the animal.

**Fig 3:**
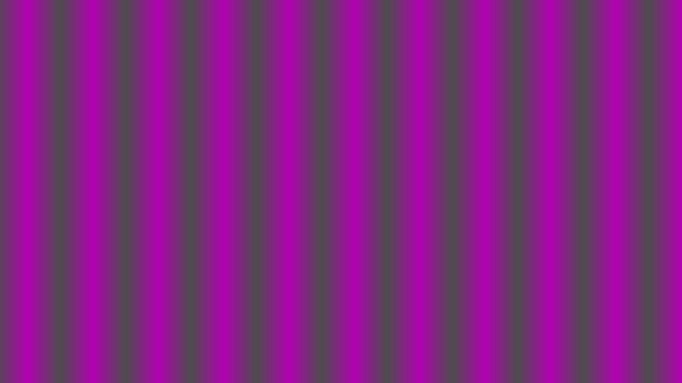
An example of sinusoidal grating pattern: Purple and black. Gratings are designed to move clockwise, anti-clockwise, or stay stationary.

In contrast to [6], our stimulation software needs no image file defining the stimulus pattern to be passed to the stimulus-presentation component. In this study, the sinusoidal gratings determined by a pair of neighboring colors (Fig 3) are applied as stimuli. Both colors can be specified by the components of the HSV (hue, saturation, value) color space, which makes it easier to test the responses of different specific color pairs. The stimulation is implemented on a laptop PC (Intel Core i7-4600M CPU, 2.90GHz, 8GB memory) with no standalone graphics processing unit (GPU). In the following experiments, the video data was recorded under the reported optimal stimulus conditions of maximum contrast, spatial frequency 0.2*cyc*/°, stimulus velocity 12°/*s* (corresponding to 2*rpm* of the proposed stimulation software) [6] [12] [16] [17].

### Region of interest

The fixation box prevents the rat from moving backwards off the platform. It partly limits the movable space in a non-contact manner. However, the rat is free to rotate its head in the three dimensions of pitch, yaw, and roll. Since the rat can only reach a limited region and the remaining captured area is not of interest, the body extraction can be simplified by selecting a region of interest (ROI). In this research, ROI is set up manually by an operator: after configuring the setup of the platform, and the camera, ROI can be defined by the coordinates of a rectangle region covering any possible positions the animal can reach. eg. (205, 131, 237, 229) corresponds to (*x*, *y*, *width*, *height*). If the relative positions of camera and platform remain the same in different experiments, it will be unnecessary to readjust the ROI.

### Image processing

To investigate the visual acuity of nocturnal rodents, the stimulus experiments were done in a lab without any light sources, except the visual stimulus. Since the stimulus pattern was designed to contain any possible color, the body-extraction algorithm should be robust to illuminance color changes. The desired body-sensing result should include not only where the body is located in the image, but also its precise contour and the location of the nose or ears.

The acquired videos were converted into image sequences, which were analyzed separately. To comprehend the current status of each image, a body-sensing module was developed to detect the body/head movement. Because the virtual cylinder only rotates on the horizontal plane, we focused on the horizontal head gaze movement of the tested subject, and the camera was set vertically. The movement was analyzed by examining the head orientation in each frame of the acquired video, which requires the head orientation in each frame to be extracted separately. Several image-processing steps are applied as described below.

To find the optimal parameter of the proposed method, either the mean squared error (MSE) or the standard deviation of determination errors can be used as the measure. The determination error refers to the error made by the proposed rat head gaze determination system while comparing the results with the ground truth. Let $\hat{Y}$ denote a vector of *m* predictions of rat head angles for a random sample, which is also the determination result by proposed method. The *Y* is the vector of ground truth data corresponding to the predictions. As shown in Eq (1), the MSE represents the difference between the determination results by the proposed method and the ground truth. The parameter value, which minimizes the MSE, obtains the smallest difference and makes the curve of determination results closest to the ground truth.

$$\begin{array}{c} \text{MSE}=\frac{1}{m}\sum_{i=1}^{m}{\left({\hat{Y}}_{i}-Y_{i}\right)}^{2} \end{array}$$ (1)

The standard deviation is a measure of the dispersion of a collection of values. In this case, the standard deviation of determination errors *σ* is given in Eq (2), where *E* denotes the vector of determination errors and *μ* denotes its mean.

$$\begin{array}{c} \sigma =\sqrt{\frac{1}{m}\sum_{i=1}^{m}{\left(E_{i}-\mu \right)}^{2}}\;,\text{where}\;E_{i}={\hat{Y}}_{i}-Y_{i}\;,\;\mu =\frac{1}{m}\sum_{i=1}^{m}E_{i} \end{array}$$ (2)

The parameter value, which minimizes *σ*, makes the data points tend to be the closest to the mean. The MSE should be used for the smallest difference from the ground truth. The standard deviation should be used to measure the variability of determination errors. In this paper, we used the MSE as the criterion to find optimal parameters. The standard deviation of determination errors is used to demonstrate the property and the performance of determination system.

#### Gamma correction

To address the problem of darkness in the recording environment, gamma correction, also known as power transformation, was utilized to adjust the intensity values by taking the original image to varying powers. As shown in Eq (3), the input image *I*_*in*_ is raised to the power $\frac{1}{\gamma }$ to get the output image *I*_*out*_. Compared with the original image acquired by the camera, gamma correction is able to lighten the dark regions and make the boundary of rat body clearer.

$$\begin{array}{c} I_{out}={I_{in}}^{\frac{1}{\gamma }} \end{array}$$ (3)

**Fig 4:**
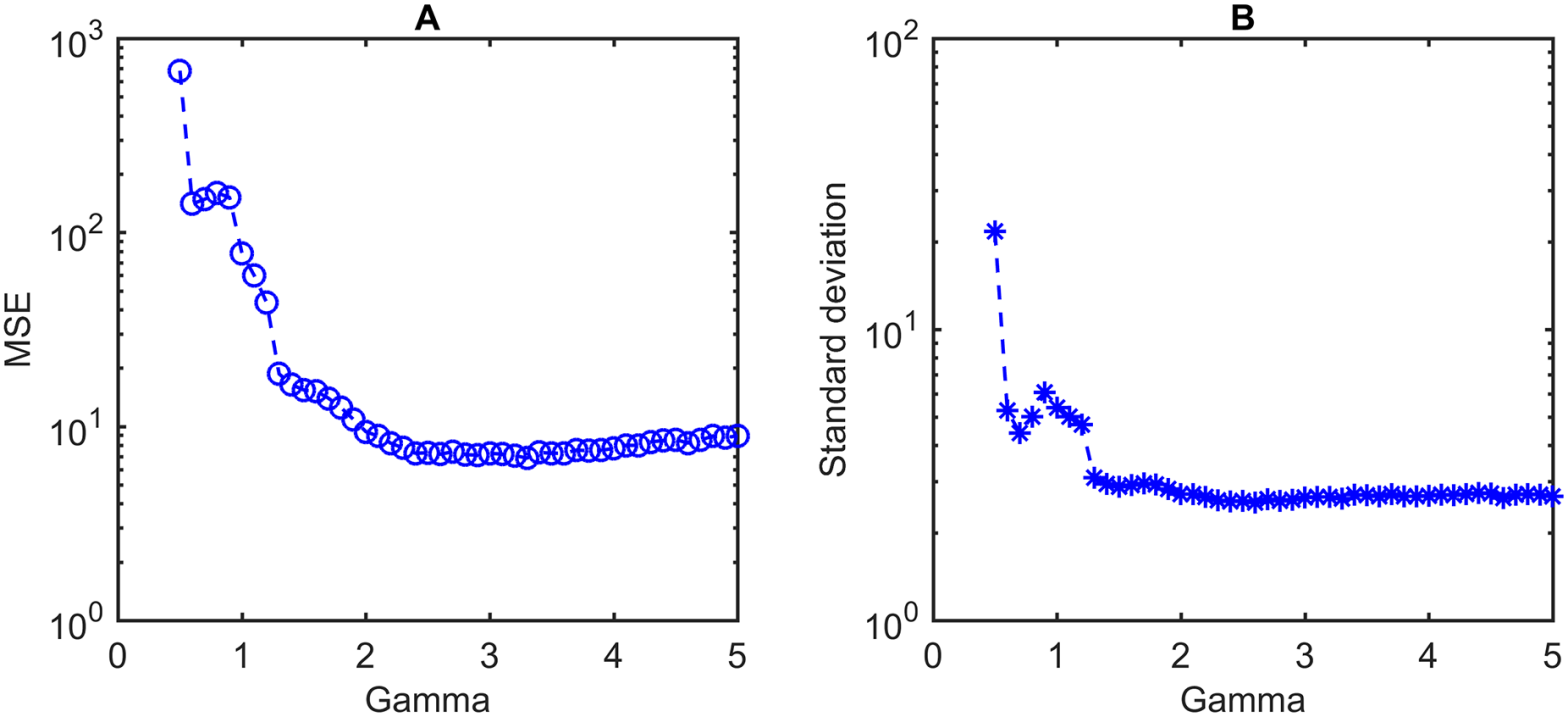
Optimization of *γ* for gamma correction. A: MSE of determination results by the proposed method. B: Standard deviation of determination error. This experiment operates on a dataset with ground truth by manual annotation. It contains 1500 captured images with head rotations over three dimensions of pitch, yaw, and roll. The *γ* to minimize MSE is 3.3, while the saturation of standard deviation is reached at 2.6. This paper use the value of 3.3 as the optimal *γ* for following experiments.

Fig 4A and 4B display MSEs and the standard deviations of determination error respectively when *γ* varies within an acceptable range. The following experiments apply the value of *γ* optimized by the measure of MSE.

#### Homogeneous filtering

The hair of rats or mice, especially whiskers or vibrissa, appears as noise that severely affects the extraction of the body contour. Approximately 30 macro vibrissae are located on each side of the face, with lengths up to 50 mm in laboratory rats, 30 mm in laboratory mice [18]. Although they cannot be clearly identified through the upright camera in dark conditions, there is a high probability that they will be classified as parts of the body, contrary to our desires. Therefore, homogeneous filtering is applied to handle this problem. It convolves the image with a simple kernel known as Normalized Box Filter, which averages the neighborhood around a pixel and assigns that value to itself. Experimentally, this kernel worked better in our scheme than the Gaussian, Median or Bilateral kernels. The kernel size is an important parameter for determining whether noises to be eliminated or interested details to be blurred.

**Fig 5:**
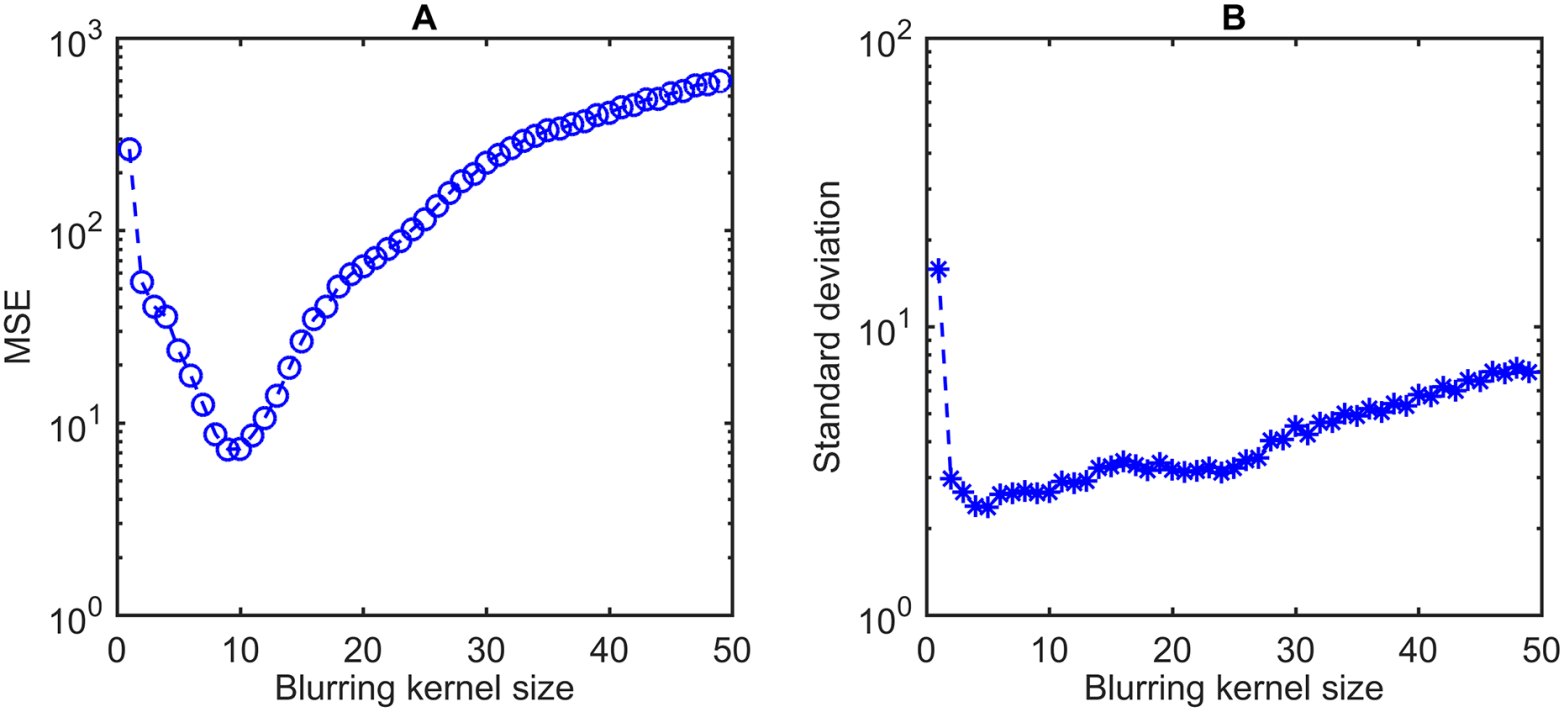
Optimization of blurring kernel size. A: MSE of determination results by the proposed method. B: Standard deviation of determination error. Blurring kernel size here means the side length of the square kernel. This experiment operates on a dataset with ground truth by manual annotation. It contains 1500 captured images with head rotations over three dimensions of pitch, yaw, and roll. The minimum of MSE is reached at 9, while the saturation of standard deviation is reached at 5.

In this paper, a homogeneous filter with the optimal kernel of 9 × 9 is adopted on images with resolution of 640 × 480. Fig 5 illustrates the optimization experiments using MSE and the standard deviation of the difference between determination results and the ground truth.

#### Binarization

Kretschmer et al [19] defined a dual threshold to perform binarization. To minimize the lighting influence, the threshold was only considered on the hue channel of the HSV or HSL (hue, saturation, lightness) color space, which is independent of the light intensity. However, it requires homogeneously colored image regions, which do not apply to markerless approaches. In this study, binarization is performed based on the intensity, and the thresholds are dynamically adjusted for frames with different lighting conditions.

**Fig 6:**
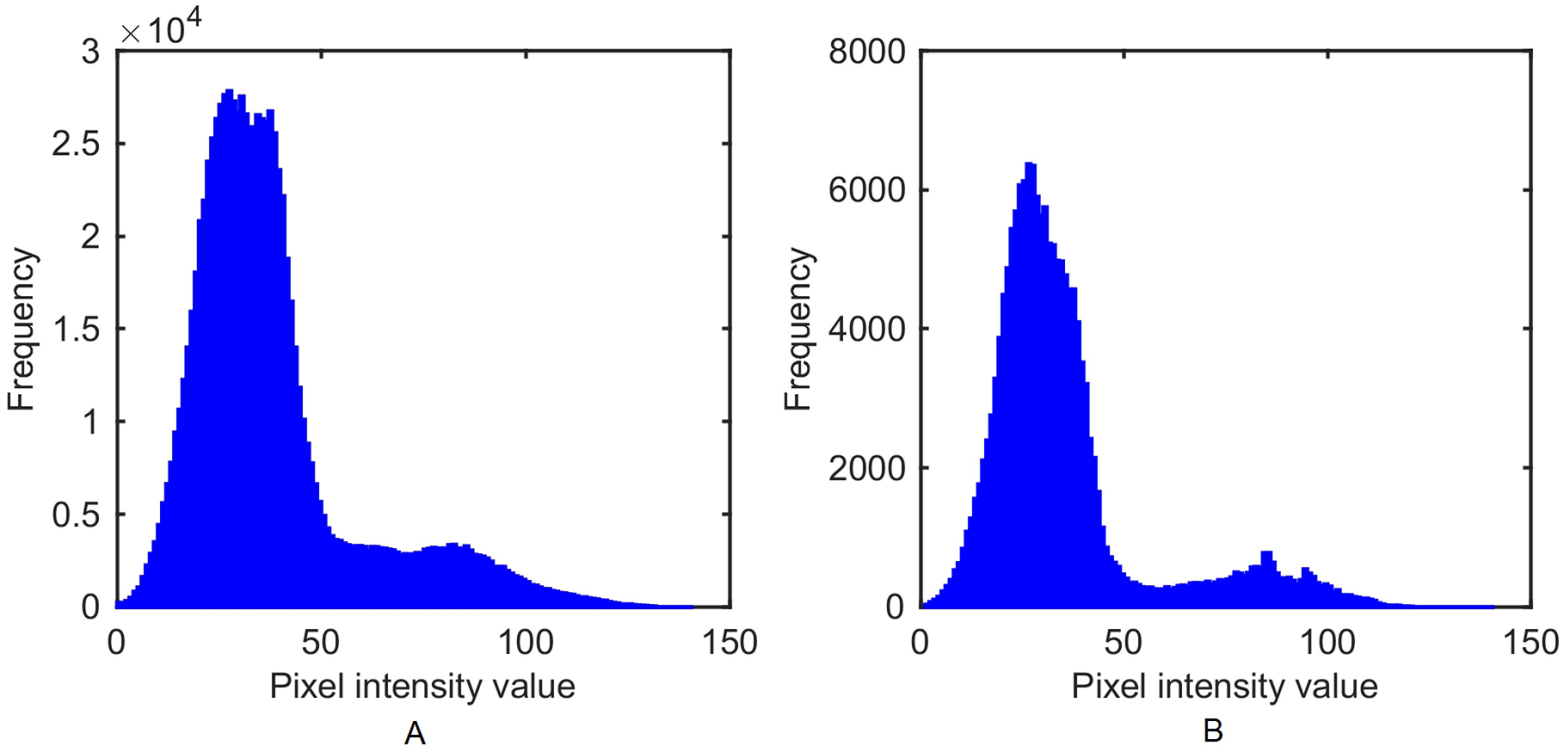
Comparison of pixel intensity value distribution on the entire image and ROI. A: Histogram of the entire image. B: Histogram of the ROI. Since all the images are captured under similar light conditions, this comparison is based on a single frame as an example.

The pixel-intensity value distribution of either the whole image (Fig 6A) or the ROI (Fig 6B) can be considered as a bimodal distribution with a deep, sharp valley between two peaks. Furthermore, the ROI distribution has a clear boundary, that makes the two peaks more separable than in the entire image. Otsu’s method [20] was used to automatically reduce a gray-scale image to a binary one, assuming that the image contains two classes of pixels, foreground pixels (body region) and background pixels [21]. The optimum threshold separates the two classes by minimizing intra-class variance, or maximizing inter-class variance, which is similar to Fisher’s Discriminant Analysis (FDA).

**Fig 7:**
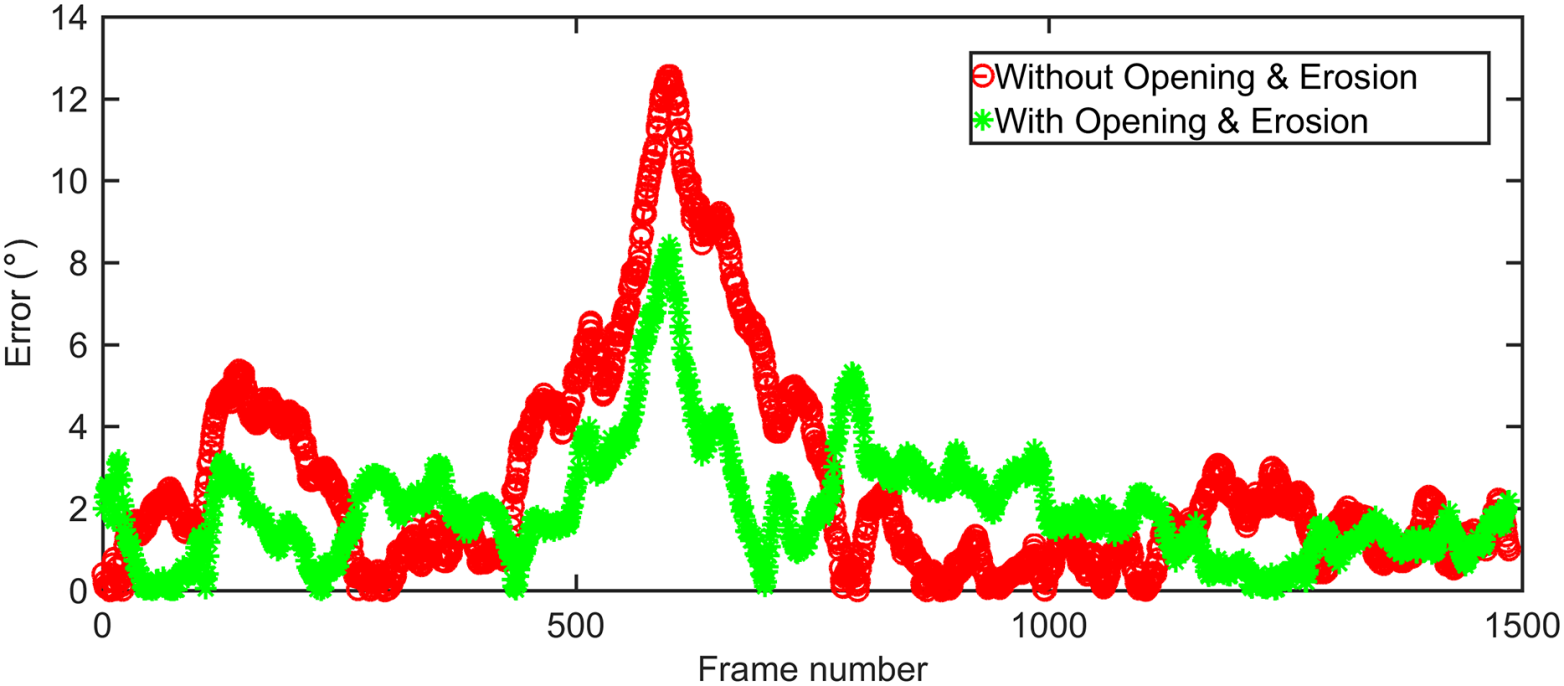
Performance with Opening & Erosion versus without Opening & Erosion. This experiment operates on a dataset with ground truth by manual annotation. It contains 1500 captured images with head rotations over three dimensions of pitch, yaw, and roll. The red curve is the determination error with Opening & Erosion, with MSE of 6.73. The parameters are optimized separately: kernal of 3 × 3 and iteration times of 3 for Opening; kernal of 3 × 3 and iteration times of 2 for Erosion. The green curve is the determination error without Opening & Erosion, with MSE of 14.45.

Morphological operations, e.g., Opening and Erosion, can mitigate the hair effects and smooth the boundary of rat body. Considering that sometimes the vibrissae and the protruding region of ears might affect the determination results of head angle, the morphological Opening followed by a Erosion process has been proved useful in reducing determination error (Fig 7). The noise regions without contiguous area to the animal body can be eliminated by identifying the candidate with the largest area as the body.

#### Contour extraction

**Fig 8:**
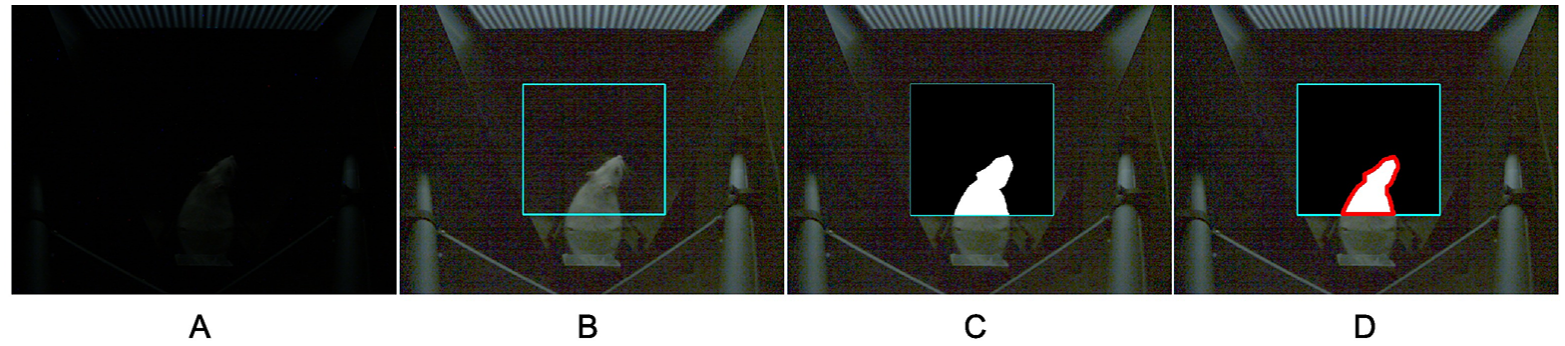
Illustration of image preprocessing steps. A: Original image (Resolution: 640 × 480). B: Gamma correction (*A* = 1.0, *γ* = 3.3; The highlighted rectangle denotes the manually selected ROI. C: Binarization (threshold detected automatically with Otsu’s method). D: Contour extraction. The red boundary is the extracted contour.

The contour is retrieved by finding the curve along the extreme outer boundary of a white object with a black background. To ensure the shape-analysis precision, we applied a contour-approximation method that stores all the contour points and demands any two subsequent points (*x*_1_, *y*_1_) and (*x*_2_, *y*_2_) to be either horizontal, vertical, or diagonal neighbors (Eq (4)). Fig 8 displays an example image in different processing steps to extract the contour within the ROI.

$$\begin{array}{c} \left\{ \begin{array}{c} |x_{1}-x_{2}|⩽1\\ |y_{1}-y_{2}|⩽1 \end{array}\right. \end{array}$$ (4)

### Head gaze extraction

A traditional head-tracking concept includes attaching artificial markers to the head of the animal. The markers help obtain the precise position of the nose, eyes, or ears. The head-tracking problem is easily solved by analyzing the markers’ position changes. However, the attached markers can distract the tested subject and affect its behavior [19].

Kretschmer et al [6] has proposed an automated head-tracking algorithm for their visual-threshold measurement system. They calculate the center of gravity in the weighted and segmented body region, and determine the nose position by finding the largest distance from the body center. Similarly, a circular masking is applied around the nose and another center of mass is calculated and determined as the head center. The head gaze vector is drawn from the head center to the nose.

As a 2D geometric concept, the curvature is the amount by which a curve deviates from being straight. The oriented curvature defines a vector that takes into account the direction of the bend, as well as its sharpness. Typically, the coordinate belonging to the body contour with the largest curvature, calculated with a proper sampling rate can be identified as the nose position. Normally, the curvature of a point on the curve is calculated using two direct neighboring points. In this paper, we propose a novel tracking algorithm based on the extracted body contour. The following algorithm is applied for each frame.

In this study, a re-sampling process is performed for points on the contour. The sampling rate means that for each point, two neighboring points are selected with a certain step length to calculate the curvature. The nose point is located at the sharpest bend relative to the overall variation tendency. Therefore, the step length should be large enough to prevent mismatches of a local sharp bend. If the step length is too small, the point with the largest curvature will be located at small bulges of the curve, such as the points around the ears.

The curvature at a point *M* is a measure of how sensitive the tangent line is to moving the point to another nearby point. Accordingly, Δ*α* denotes the tangent-angle deviation and Δ*s* denotes the arc-length deviation. The scalar curvature *k* at point *M* is given by

$$\begin{array}{c} k=\lim_{\Delta s\to 0}|\frac{\Delta \alpha }{\Delta s}| \end{array}$$ (5)

For curves that can be expressed by *y* = *f*(*x*) and are twice continuously differentiable, the scalar curvature can be calculated with

$$\begin{array}{c} k=\frac{\left|y^{\prime \prime }\right|}{{\left(x+y^{\prime 2}\right)}^{\frac{3}{2}}} \end{array}$$ (6)

In this study, suppose the extracted contour *C* is twice continuously differentiable and there exists a parametric representation of *C* by a pair of functions:

$$\begin{array}{c} \left\{ \begin{array}{c} x=\varphi (t)\\ y=\psi (t) \end{array}\right. \end{array}$$ (7)

Then,

$$\begin{array}{c} k=\frac{\left|\varphi^{\prime }\left(t\right)\psi^{\prime \prime }\left(t\right)-\varphi^{\prime \prime }\left(t\right)\psi^{\prime }\left(t\right)\right|}{{\left[\varphi^{\prime 2}\left(t\right)+\psi^{\prime 2}\left(t\right)\right]}^{\frac{3}{2}}} \end{array}$$ (8)

**Fig 9:**
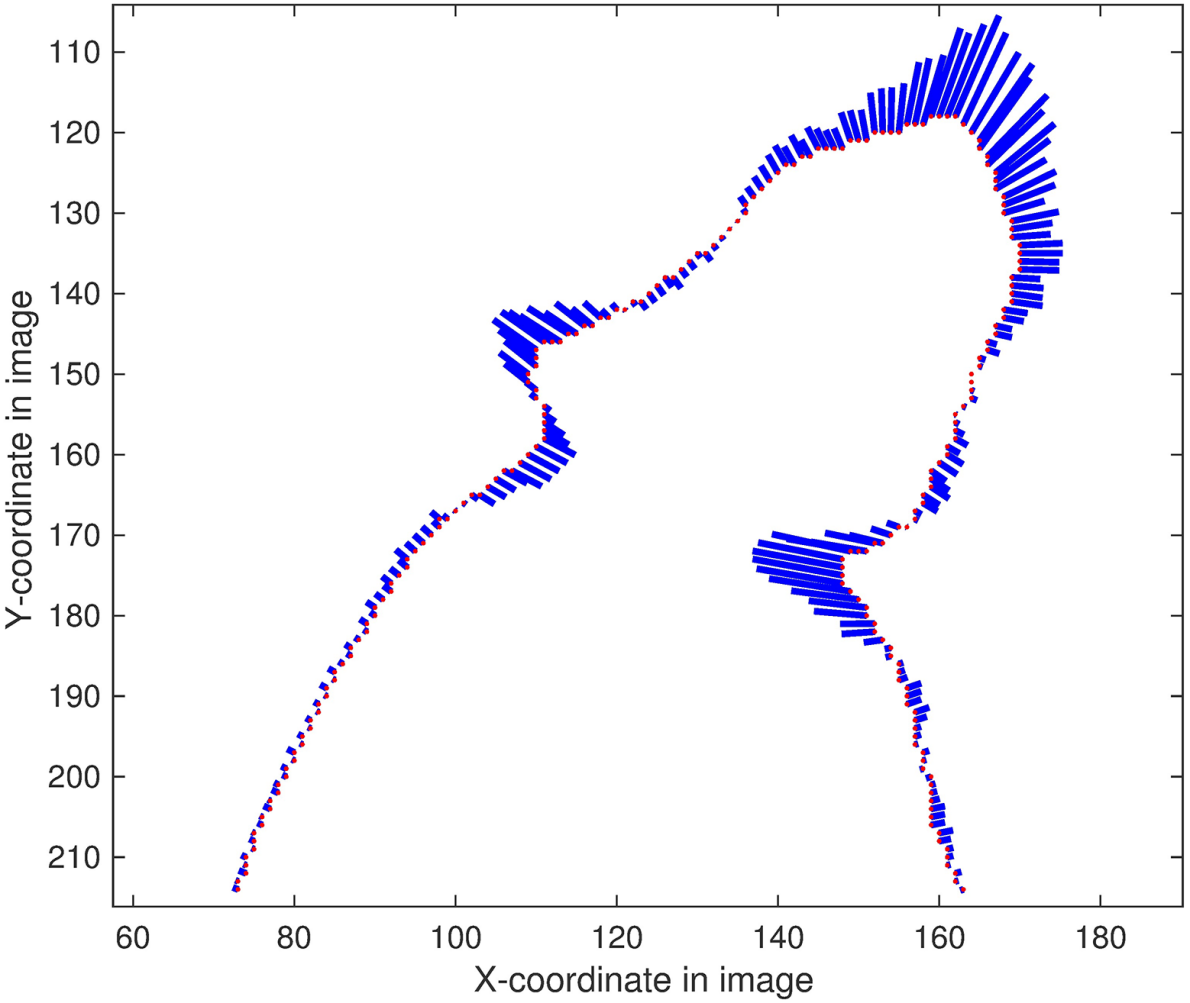
Curvatures and normals of the points on the contour. Step length of neighboring points: 16.

Fig 9 displays the scalar curvatures and normals of each point on the contour. Each blue vector has the same orientation as the normal line and the same magnitude as the curvature. The sharp turning points have larger curvatures than the flat segment points. The nose position is considered as the point with the longest vector.

The head gaze is defined by the vector from a point on the head’s symmetrical center line to the nose. The start point is set by the mid-point of two symmetrical points on the head segment of the contour. This pair of symmetrical points can be found because they share the same distance to the nose along the boundary. The position of the nose relies on the curvature at the points on the contour. However, calculating the curvature for all contour points of all frames in the video is fairly time-consuming. A simplified algorithm (Algorithm 1) is presented to rapidly retrieve the head gaze vector for each image:

**Algorithm 1** Head Gaze Vector Extraction

1: **inputs**:

 *C* = {*P*_1_, *P*_2_, *P*_3_, …*P*_*N*_}      ▹contour with *N* sorted points

 *stepRatio*      ▹ ratio of step length between sampled points to contour length

2: **initialize**:

 *step* ← *N* × *stepRatio*, *i* ← 1 + *step*, *minIncludedAngle* ← 360°, *nose* ← 0

3: **while** *i* − *step* ≥ 1∨*i* + *step* ≤ *N* **do**

4:   *P*_*index*_ ← *P*_*i*_      ▹assume current point as nose

5:   *P*_*left*_ ← *P*_*i*−*step*_      ▹generate left neighbor

6:   *P*_*right*_ ← *P*_*i*+*step*_      ▹generate right neighbor

7:   *α* ← ∠*P*_*left*_ *P*_*index*_ *P*_*right*_     ▹compute included angle

8:   **if** *α* < *minIncludedAngle* **then**

9:    *minIncludedAngle* ← *α*

10:    *nose* ← *i*

11:   **end if**

12:   *i* ← *i* + 1

13: **end while**

14: *P*_*mid*_ ← (*P*_*nose*−*step*_ + *P*_*nose*+*step*_)/2      ▹compute midpoint of two neighbor points

15: **return** $\vec{P_{mid}P_{nose}}$

**Fig 10:**
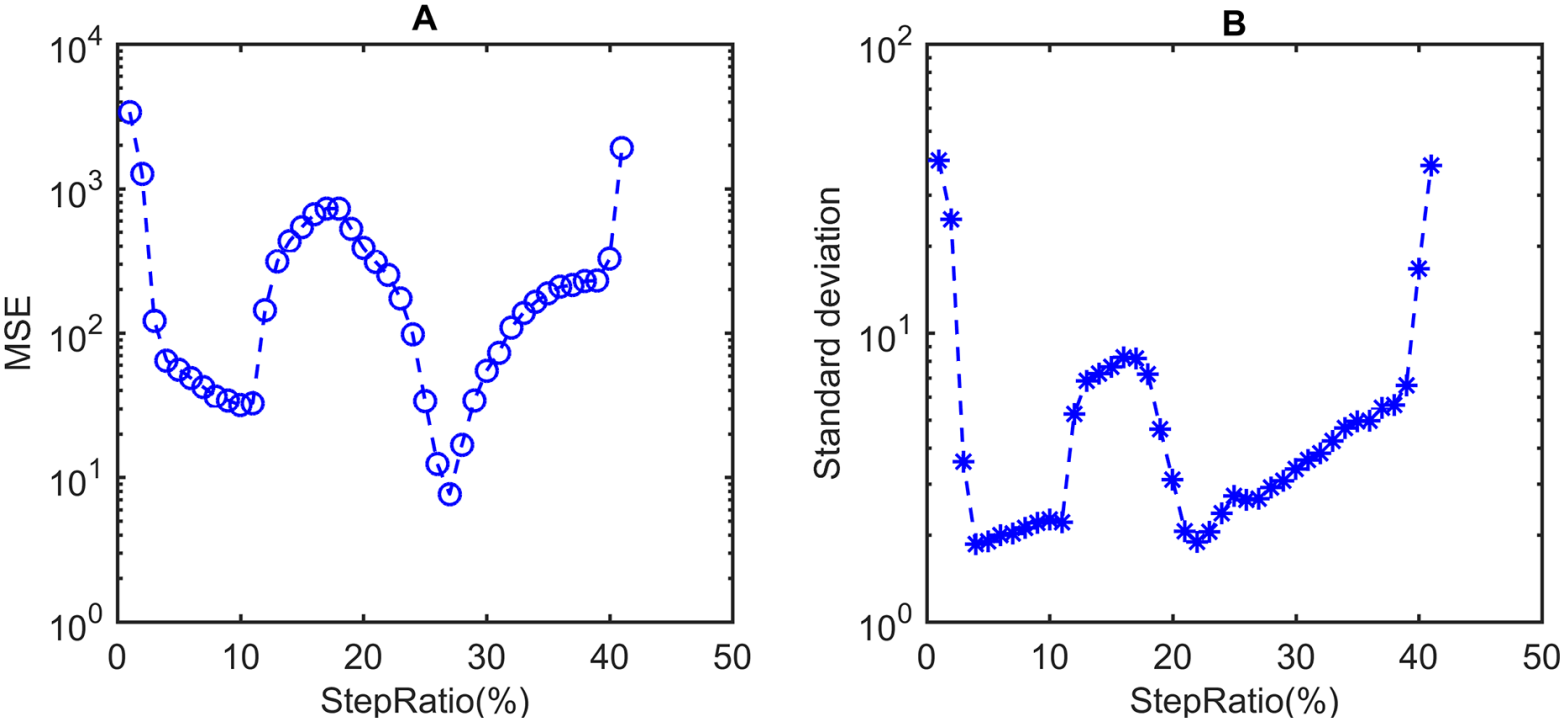
Optimization of *stepRatio*. A: MSE of determination results by the proposed method. B: Standard deviation of determination error. This experiment operates on a dataset with ground truth by manual annotation. It contains 1500 captured images with head rotations over three dimensions of pitch, yaw, and roll. The *stepRatio* to minimize MSE is 27%, while the saturation of standard deviation is reached at 4.

The parameter of *stepRatio* is a percentage of the number of contour points which is applicable to rats of similar body size and head/body ratio. The intersection between the bottom line of the ROI and the closed polygon (red boundary in Fig 8D) has been included in the contour point set, which has been proved to provide a better step length. Fig 10 demonstrates the process of optimizing *stepRatio* with help of ground truth.

## Results

### Output

**Fig 11:**
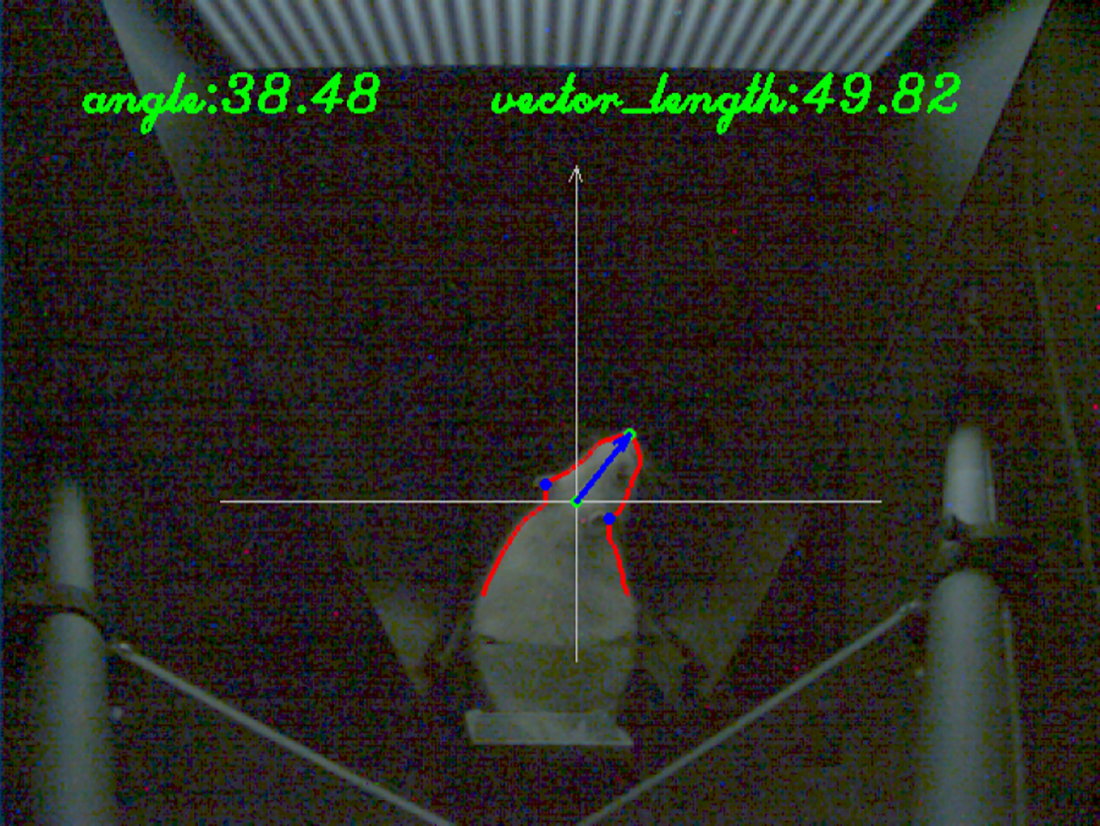
An example of the head gaze determination results. The red line is the extracted body contour within the ROI. The blue arrow is the extracted head vector with the aid of four supporting points: two symmetrical points on the contour (in blue), their mid-point (in green) and the nose point (in green). All the points, lines, and text in this example image are automatically generated by the implementation program of the proposed method.

The head gaze orientation is examined by the angle included between the head gaze vector and the vertical line. The vector length decreases considerably when the animal tilts its head upwards or downwards, which can be used to make improper frames invalid for analysis. An example of the output image is shown in Fig 11. Besides this image, the implementation program also outputs another text file recording information of the original image, extracted head angle, and length of head gaze vector.

### Evaluation

To evaluate the performance of the automatic head gaze extraction, a ground-truth data set was made by manually selecting at the positions of the nose and a pair of symmetric eye points. A small software tool was developed to assist in the manual operation and automatically calculate the head gaze angle with coordinates of three points. We randomly selected a clip from the compact videos, which contains 1500 frames and the compact video data contains about 6000 frames. The clip was assigned to an operator to generate the ground-truth data following steps as bellow frame by frame:

- Click on the nose position to save the nose coordinates.
- Click on a pair of symmetric points around both eyes to save their coordinates.
- The head gaze vector is calculated and saved automatically.

**Fig 12:**
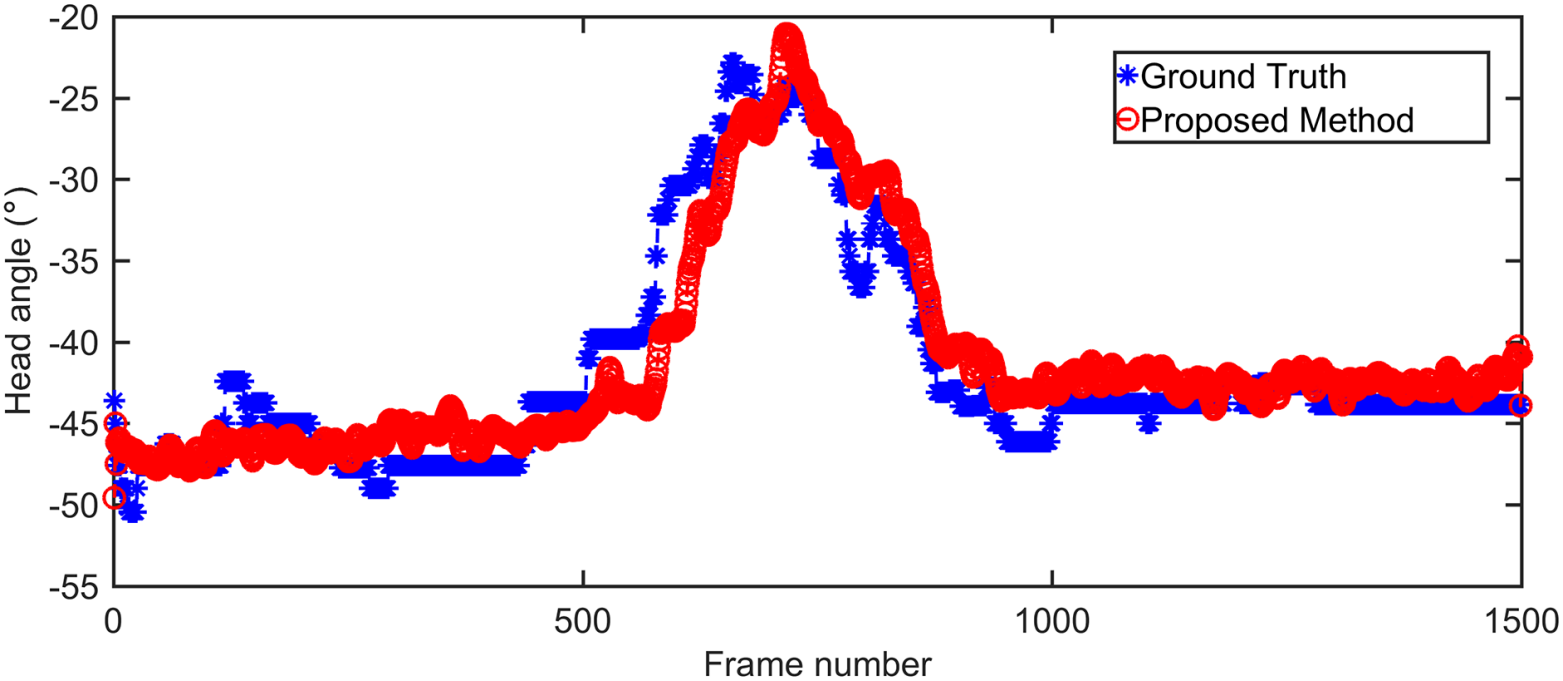
Determination results of head angle between proposed method and ground truth. This experiment operates on a dataset with ground truth by manual annotation. It contains 1500 captured images with head rotations over three dimensions of pitch, yaw, and roll. The red curve is the optimal determination results with our proposed method, with MSE of 6.73. The blue curve is the ground truth by manual annotation.

The image is enlarged (×2) while clicking to obtain the precise coordinates. However, the operator must conjecture when the nose or eye cannot be seen from the camera. Therefore, it is still difficult to avoid uncertainty by the judgment of human beings. As shown in Fig 12, by optimizing all adjustable parameters statistically, the determination results by our proposed method are optimal with only a systematic small delay to the ground truth.

### Illustration of stimulus tracking behavior

**Fig 13:**
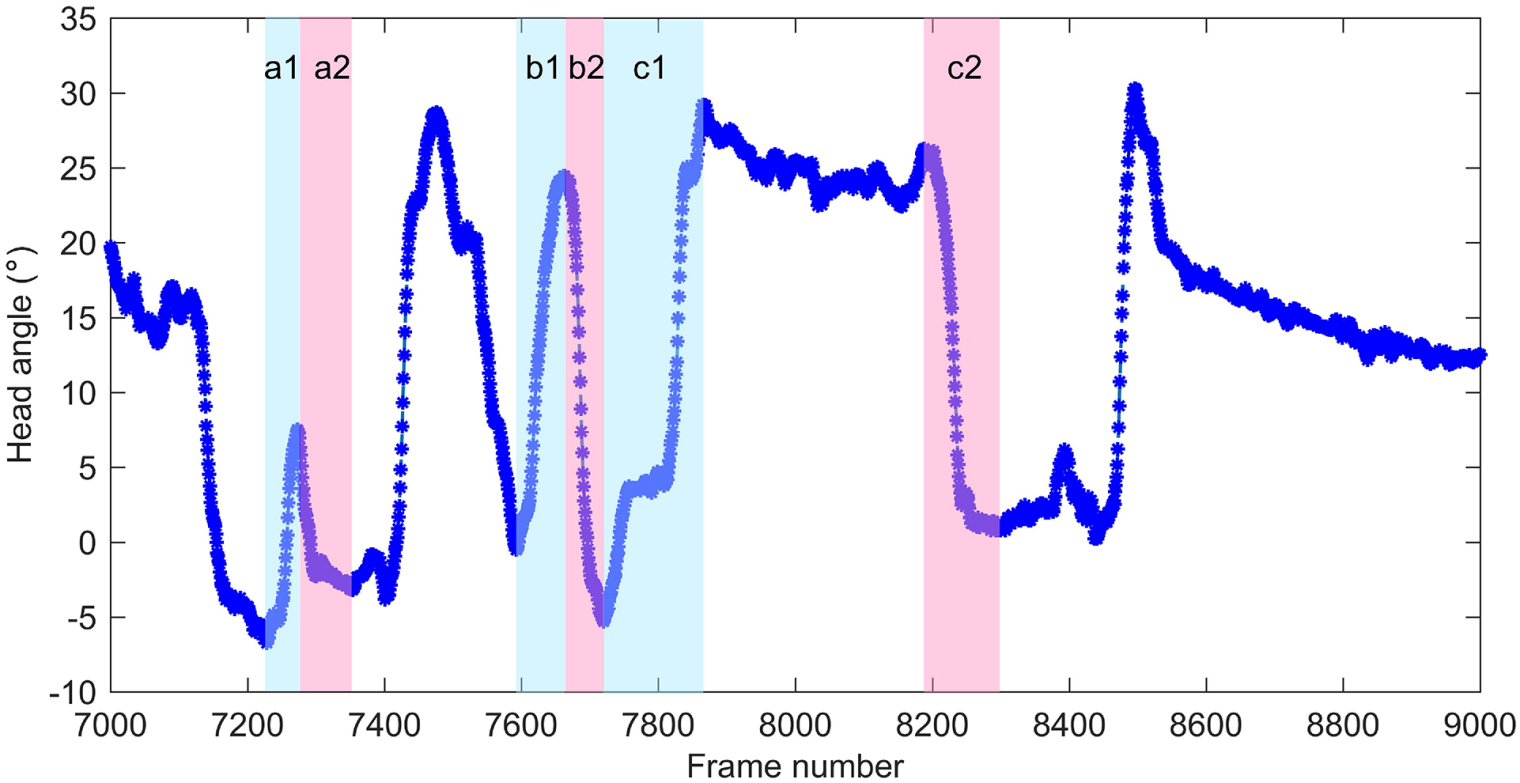
Illustration of head movement in stimulus direction and anti-stimulus direction. The curve of head angles over 2000 frames is part of the determination results of a set of video data. The video data is saved at 30fps and the stimulus velocity was 12°/*s*.

The stimulus tracking behavior is defined by frames in which the head turning direction is same to the movement of stimulus. Fig 13 illustrates the head movement in stimulus direction and anti-stimulus direction. According to this figure, head movement in stimulus direction is always followed by corresponding head movement in anti-stimulus direction (Phase *a*2 and *b*2) or a stable phase (phase between *c*1 and *c*2). Each phase of continuous head movement in stimulus direction maintained a steady average velocity: 10.57°/*s* in Phase *a*1; 11.47°/*s* in Phase *b*1; 5.96°/*s* in Phase *c*1. Both Phase *a*1 and *b*1 shares similar average velocity to the stimulus. Phase *c*1 had much lower velocity because of the existence of a short stable phase within *c*1. On the other hand, continuous head movement in anti-stimulus direction have varied average velocity, and they ended with a sudden slow-down (Phase *a*2), a reverse movement (Phase *c*1), or a stable phase (Phase *c*2).

## Discussion

### Mouse versus rat

Rats and mice are rodents with similar appearances, but are obviously differentiated by body size. Even baby rats are larger than adult mice. However, they have so many similar physical characteristics that unprofessional observers cannot tell them apart by their pictures. Therefore, we believe it may be possible to use the same algorithm to analyze the body shape for both rodents, based on captured images. Morphologically, mice often have smaller heads and larger ears and eyes relative to the head than rats. The proposed automated body-sensing algorithm can theoretically be applied to both rats and mice. However, the experiments in this study were done on rats.

### Movement restriction

In a strict sense, the movement of rat is not actually restricted in our setup: the body of the rat is neither attached with tapes, nor blocked from moving. The fixation box is large enough for the rat to move forwards freely and turn its head around freely. The fixation box only performs as a small room to limit its movable space and ensure that the virtual cylinder of the stimulus is centered at the head. The detected head angle with a larger value than 45° is determined as the frame that rat turns its head away from the stimulus. These frames are made invalid in the analysis of optomotor responses. When the rat moves its head away from the pre-defined center of the virtual cylinder, the stimulus is not able to maintain a constant shape from the visual angle of the rat. This situation is ignored in this study because only a small range ([−45°, 45°]) of head turning is accepted as a valid response to stimulus.

### Comparison with existing methodologies

As a markerless system, our tracking algorithm has advantages over marker based systems, e.g., no distraction from artificial markers and no front/back ambiguity. Compared with the previously available markerless approaches proposed in [19], the drawback of our apparatus is that the lower body is restricted into a fixation box and only the upper body can move freely. Consequently, the visual range is also limited to about 90°. However, our experiments proved that the apparatus can detect the stimulus tracking movements of rat despite these restrictions. Since frames with inclinations on the vertical plane are eliminated by thresholding the length of head gaze vector, our algorithm will not be influenced by the lateral inclination on the horizontal plane.

Another apparatus to measure the optomotor response proposed by Kretschmer et al. [16] used LED strips attached in all four edges and mirrors on both bottom and top side of their setup to provide gratings inside a virtual sphere surrounding the animal. The top camera captures the animal, a small part of the platform supporting the animal, a large square area of the bottom mirror reflecting gratings on four computer screens. However, the body contours were not extracted correctly when there is no clear boundary between the body region and the background. The performance of their methodologies will be influenced by the light conditions, the color of gratings, and the coat color. In our research, the apparatus is designed to provide the grating stimulus with a yaw range of 90°. The bottom and surrounded panel can be replaced by materials with less refection to reduce noise while detecting contour. In this paper, we used metal panels of dark colors to make a clear color difference between the animal region and other regions. For non-albino animals, the panels can be replaced by other materials of different color and absorption properties, corresponding to the coat color. By this way, our solution is supposed to detect animal contour of different coat colors successfully. However, we have not tested any non-albino animals in experiments of this paper, which will be part of our future work.

In the setup introduced by Kretschmer et al. [16], the stimulus was presented on four monitors. The camera operated on the frequency of 240Hz in their setup. By using a projector-based setup in our setup, the maximum capturing frequency was 100Hz, and it was proved to be enough to extract correct contours and determine head gaze of the animal by our algorithm. The capturing frequency of our setup is less than half of that of previous literature. Under the premise that the system is able to determine head gaze precisely, our projector-based setup is much cheaper and easier to prepare. Furthermore, the screen size for the projector is easily changeable according to different requirements. We did not check the difference of light wavelength between a projector screen or a monitor, because the purpose of this research is to develop a robust solution to determine rat head gaze for automatic processing of massive captured data in the optomotor response experiments. In the proposed scheme, using either a sole monitor or a sole projector screen to present stimulus does not change the color histograms of ROI and will have little influence on the results of head gaze determination.

### Future plans

A previous literature [6] used a tolerated range (±9°/*s*) of the head angular velocity centered at the stimulus velocity as stimulus tracking behavior. Since in our apparatus, rat is put on the platform and surrounded by the fixation box, which is different from the literature, the adjustment of this range of velocity is necessary, which is part of our future plan. Furthermore, the distraction caused by the apparatus also requires quantified measurement in the future.

Our future work will focus on synchronizing the stimulus and the head movement to guarantee that the virtual stimulus cylinder is always centered at the detected head center. Additionally, we will design a similar but smaller fixation box for mice and perform automatic head gaze determination for OMR experiments.

## Conclusion

We developed a novel measurement system to automatically extract a rat’s head gaze vector for each frame in a video clip. The framework proposed in this study was derived from the commercially available visual optomotor system, OptoMotry, but was distinguished by two key contributions. Firstly, we utilized a projector and a screen to present the stimulus. The projector can produce a higher illuminance than computer monitor and make the measurements more sensitive to rodents with poor vision or gradual vision loss. The screen size for the projector is easily changeable according to different requirements. Secondly, to our knowledge, this is the first time that the body contour is utilized to calculate the head gaze vector. The recent literature [16] extracted contour to obtain body region. However, center of gravity, rather than contour points, is used to calculate head gaze, in accordance with the study by Kretschmer et al. [6]. The proposed method applied body contour originally and successfully to head gaze determination. This paper also is the first time to make use of ground truth data for parameter optimization and performance estimation. The comparison between results of proposed method and ground truth proved that our method provides a simple and reliable scheme to automatically determine the head gaze. Moreover, according to statistics of randomly selected 6000 frames, the average processing speed was as fast as 51.79fps on a desktop PC (Intel i5-3570 CPU, 3.40 GHz, 8 GB memory).

### Ethics statement

All experimental procedures were approved by the Institutional Animal Care and Use Committee of Iwate University (No. A201505). All animal experiments were conducted in accordance with the guidelines of the Animal Experiment Committee of Iwate University, Japan. Wistar rats were obtained from CLEA Japan, Inc. (Tokyo, Japan). The Wistar rats were kept in cyclic light (12 h on/off) ad libitum with free access to water. Rats were not sacrificed in the view of ethic of animal protection and kept for other studies.

## Supporting information

S1 Video The movie of automatically detected rat head gaze vectors. The original video was recorded by 100fps. Our head gaze determination system processed the recorded video frame by frame and outputted this movie with result information for each frame. Four digits on the upper left corner showed the included angle (°) between the vector and the vertical axis. Clock-wise was defined as positive. Four digits on the upper-right corner showed the length in pixels of the vector. The movie was transcoded and compressed with the frame rate of 30fps.
(MP4)

S1 File Experimental data file. All the experimental data is also available online [22]. This file compressed images, contour point coordinates, and head angle record files supporting experiments of this study, e.g. binarization analysis, curvature analysis, tangent angle analysis, head angle detection evaluation, optimization experiments for parameters of blurring kernel size, *stepRatio* in proposed algorithm, *γ* in gamma correction, and the validation of opening and erosion.
(ZIP)
