# Supplementary figures and images for: A novel rat head gaze determination system based on optomotor responses
Source: PLoS One. 2017 Apr 26;12(4):e0176633. doi: 10.1371/journal.pone.0176633 (PMC5405982; doi:10.1371/journal.pone.0176633)

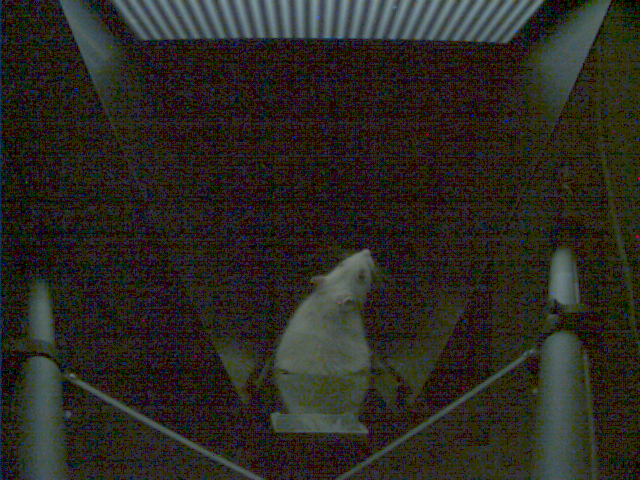

Supplement: S1 File — All the experimental data is also available online [22]. This file compressed images, contour point coordinates, and head angle record files supporting experiments of this study, e.g. binarization analysis, curvature analysis, tangent angle analysis, head angle detection evaluation, optimization experiments for parameters of blurring kernel size, stepRatio in proposed algorithm, γ in gamma correction, and the validation of opening and erosion. (ZIP) [file pone.0176633.s002.zip › 1-binarization-analysis/gamma.bmp]

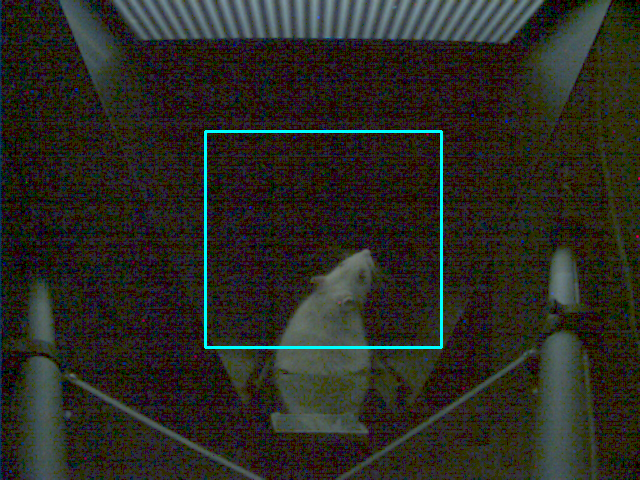

Supplement: S1 File — All the experimental data is also available online [22]. This file compressed images, contour point coordinates, and head angle record files supporting experiments of this study, e.g. binarization analysis, curvature analysis, tangent angle analysis, head angle detection evaluation, optimization experiments for parameters of blurring kernel size, stepRatio in proposed algorithm, γ in gamma correction, and the validation of opening and erosion. (ZIP) [file pone.0176633.s002.zip › 1-binarization-analysis/roi.bmp]
